# Beyond Conventional Autopsy: Recent Advances in Molecular Autopsy for Sudden Cardiac Death Investigation

**DOI:** 10.3390/genes17101300

**Published:** 2026-10-19

**Authors:** Cecilia Salzillo, Andrea Marzullo

**Affiliations:** Pathology Unit, Department of Precision and Regenerative Medicine and Ionian Area, University of Bari “Aldo Moro”, 70124 Bari, Italy; andrea.marzullo@uniba.it

**Keywords:** molecular autopsy, sudden cardiac death, forensic genetics, next-generation sequencing, variant interpretation

## Abstract

Sudden cardiac death (SCD) is a leading cause of unexpected mortality worldwide and represents a major challenge in pathology, especially when conventional autopsy fails to identify a definitive cause of death. In these cases, molecular autopsy (MA) has emerged as a valuable diagnostic approach, enabling the identification of pathogenic genetic variants associated with inherited cardiomyopathies, channelopathies, and other cardiovascular diseases. This review provides a comprehensive overview of the current role of MA in the investigation of SCD, highlighting recent advances in postmortem genetic testing and their impact on forensic practice. The evolution of sequencing technologies, from targeted gene panels to whole-exome and whole-genome sequencing, as well as new long-read sequencing approaches, is discussed. Emphasis is placed on the interpretation of genetic variants according to current international guidelines, the integration of molecular findings with autopsy, histopathological, and toxicological investigations, and the importance of multidisciplinary collaboration between forensic pathologists, molecular geneticists, cardiologists, and genetic counselors. We also examine current challenges, including variants of uncertain significance, limited genotype–phenotype correlation, ethical considerations, and the need for standardized international protocols. Finally, we explore prospects, including multi-omics technologies, artificial intelligence-assisted variant interpretation, and improved data sharing initiatives, which may improve diagnostic accuracy and facilitate clinical translation. In addition to determining cause of death, MA has become an essential tool for identifying at-risk relatives through cascade screening, thus extending the role of forensic genetics from postmortem diagnosis to preventive cardiovascular medicine.

## 1. Introduction

Sudden cardiac death (SCD) is one of the leading causes of unexpected mortality and represents a major diagnostic challenge in the clinical, anatomopathological and medico-legal fields. Although SCD is frequently associated with identifiable structural heart disease, a significant proportion, especially in young subjects (SCDY), occurs in the absence of a previous cardiovascular diagnosis and may represent the first clinical manifestation of an inherited heart disease [1,2,3]. The main pathologies responsible for SCDY include cardiomyopathies, channelopathies, aortopathies, coronary artery diseases and other genetically determined cardiovascular pathologies, some of which may not produce morphological alterations evident at conventional autopsy [2,3]. The identification of the cause of death therefore assumes not only a diagnostic and medico-legal value, but also a relevant preventive implication, since it can allow the identification of hereditary conditions in the victim’s family members [1,4].

A particular difficulty is represented by autopsy-negative SCD cases, in which macroscopic and histopathological examination do not reveal alterations sufficient to explain death. In young people, the presence of a structurally normal heart or non-specific pathological findings may suggest genetically determined conditions, such as channelopathies or early and phenotypically silent forms of cardiomyopathy [2,3,5]. In a recent study conducted on 59 subjects aged between 18 and 45 years, sudden unexplained death (SUD) was the most frequent finding, affecting 35.6% of cases [5]. Similarly, a prospective study of consecutive cases of sudden natural death highlighted that the absence of a definitive autopsy cause remains a relevant problem, especially in the young [6]. These data confirm that conventional autopsy, although representing the cornerstone of postmortem investigation, may not be sufficient to identify inherited arrhythmogenic conditions or occult cardiomyopathies.

Molecular autopsy (MA) has progressively established itself, understood as the integration of genetic analysis into post-mortem investigation aimed at identifying variants potentially responsible for SCD. Current recommendations and operational proposals support the use of post-mortem genetic testing in cases of sudden death in which the autopsy is negative or inconclusive or where a hereditary heart disease is suspected [1,4]. The evolution of next-generation sequencing (NGS) technologies has allowed us to move beyond the analysis of single genes, allowing the use of increasingly larger multigene panels and, in selected cases, approaches such as whole-exome sequencing (WES) and whole-genome sequencing (WGS) [4,7]. A 2024 study of suddenly deceased athletes highlighted the presence of clinically actionable variants in 17% of subjects undergoing MA, underlining the diagnostic potential of post-mortem genetics even in apparently healthy and physically active individuals [7].

The diagnostic yield of MA, however, is not uniform and depends on the population studied, the case selection criteria, the autopsy phenotype and the genetic strategy adopted. A study conducted in a Chinese population demonstrated the ability of MA to identify potentially causative variants and to subsequently guide cascade screening of family members [8], suggesting that yield should be interpreted in the context of the overall phenotype and not as an isolated genetic test.

A fundamental element therefore remains the correct interpretation of genetic variants. The identification of a rare variant does not necessarily imply a causal relationship with the lethal event and requires a systematic integration of genetic data with clinical, familial, autopsy and histopathological information [4,9]. Classifying variants according to shared criteria and focusing on variants of uncertain significance (VUS) are important aspects to avoid overdiagnosis or causal attributions not supported by available evidence [4,9]. Complexity increases further in cases characterized by incomplete penetrance, variable expressivity, or discordance between genotype and phenotype. Recent contributions have underlined the importance of a multidisciplinary evaluation in which the molecular data is interpreted together with the macroscopic and microscopic findings of the autopsy and the family history [9,10].

Furthermore, MA takes on a significance that goes beyond determining the cause of death of the individual victim. The identification of a pathogenic or likely pathogenic variant associated with an inherited cardiovascular disease may allow biologically related family members to be referred for targeted cardiac and genetic evaluation [1,8,10]. Cascade screening allows the identification of subjects carrying the same variant, even in the absence of clinical manifestations, thus allowing the adoption of personalized surveillance and prevention strategies [1,8]. Recent studies have also highlighted how the integration between autopsy, genetics and clinical evaluation of family members can facilitate the identification of occult cardiomyopathies and channelopathies and contribute to the prevention of further SCD events within families [8,10,11]. MA is therefore an important point of connection between cardiovascular pathology, forensic medicine, genetics and preventive cardiology.

At the same time, rapid advances in genomics are reshaping the interpretative framework of autopsy. NGS technologies expanded gene panels, WES, and WGS now enable the identification of a growing number of genes and variants, while emerging multi-omics approaches and postmortem biomarkers may further enhance the diagnostic potential of MA in the future [4,12]. However, the increasing complexity of genetic data requires standardized procedures, specialized expertise, and close collaboration among pathologists, forensic pathologists, molecular geneticists, cardiologists, and other professionals involved in SCD investigation [9,12]. Therefore, standardizing sampling, sequencing, variant interpretation, and reporting protocols remains a major challenge for the full integration of MA into medico-legal practice [4,12].

This review aims to critically analyze the most recent evidence regarding the role of MA in SCD, with particular attention to the main genetic conditions associated with sudden death, postmortem sequencing strategies and the interpretation of variants. The integration of genetic data with autopsy, histopathological, and toxicological evidence, the main diagnostic, ethical, and medico-legal issues, and the prospects of postmortem genomics will also be discussed. Finally, particular attention will be paid to the possibility of transforming the results of the post-mortem investigation into a tool for familial cardiovascular prevention, through the early identification of genetically predisposed subjects [1,8,10,13].

The clinical framework for molecular autopsy has been substantially defined by the 2020 APHRS/HRS expert consensus statement on the investigation of decedents with sudden unexplained death and their families [13]. This consensus emphasizes that molecular autopsy should be integrated into a comprehensive post-mortem investigation that includes detailed personal and family history, complete cardiovascular autopsy, histopathology, toxicology, appropriate biological sample preservation, multidisciplinary evaluation, and phenotype-directed genetic testing. Importantly, the consensus also extends the diagnostic pathway beyond the deceased individual by recommending clinical and genetic evaluation of first-degree relatives when an inherited disorder is suspected or identified.

## 2. Molecular Autopsy in Sudden Cardiac Death

MA can be defined as the integration of post-mortem genetic analysis into the diagnostic investigation of SCD, with the aim of identifying genetic variants potentially responsible for the lethal event and attributable to hereditary cardiomyopathies, channelopathies or other genetically determined cardiovascular conditions [13,14,15]. Unlike conventional autopsy, which is primarily aimed at identifying anatomical and histopathological alterations, MA also allows for the investigation of forms of the disease characterized predominantly by an arrhythmic phenotype or by molecular alterations not necessarily associated with a morphological substrate. The most recent evidence confirms that post-mortem genetic analysis constitutes an important component of the investigation in cases of SCDY, particularly when autopsy does not identify a conclusive cause or when the findings suggest a possible hereditary origin [14,15,16]. Current strategies mainly include dedicated multigene panels for cardiomyopathies and channelopathies and, in selected cases, more extensive approaches using WES or WGS [14,15,17].

The main indications for MA include cases classified as sudden unexplained death (SUD) or sudden arrhythmic death syndrome; however, MA may also be considered when autopsy reveals findings of uncertain significance or features suggestive of cardiomyopathy [8,14,18].

The choice to perform an MA should therefore be based on an integrated phenotype-guided and clinicopathological approach, rather than on the indiscriminate application of a genetic test to all cases of sudden death. The frequency with which MA should be performed in sudden death remains difficult to define, as diagnostic yield varies substantially according to age, phenotype, case selection, and sequencing strategy. Available evidence supports a selective rather than universal approach. In a recent prospective study of consecutive cases of unexplained natural sudden death in individuals aged ≤50 years, post-mortem genetic testing was routinely performed in subjects aged ≤35 years, whereas in those aged 36–50 years it was reserved for cases in which no cause of death was identified or an inherited cardiac phenotype was suspected. The positivity rate was 7.6% in subjects aged ≤35 years and 4.9% in those aged 36–50 years; when genetic testing was modelled as a systematic strategy in the older group, the estimated yield decreased to 2.5% [6]. Similarly, in a large UK registry of 748 athletes who died suddenly, only 42 (6%) underwent MA, with clinically actionable variants identified in 17% of those tested [7]. These findings support a phenotype-guided strategy in which molecular testing is considered after a complete cardiovascular autopsy, histopathological examination, and toxicological investigation, particularly in young decedents with an unexplained or inconclusive cause of death, a structurally normal heart, equivocal pathological findings, or a personal or family history suggestive of inherited cardiac disease [14,15]. The most recent operational proposals have also suggested an organization of genetic tests into first- and second-line strategies, adapted to the clinical-pathological context and to the pre-test probability of a specific hereditary condition [4].

However, MA should be considered as an extension and not a replacement of conventional autopsy. An isolated genetic result is not in fact sufficient to establish with certainty the cause of death. The presence of a variant must be interpreted considering the overall phenotype and the biological and clinical plausibility of the relationship between the variant and the disease [14,15]. In particular, the simple identification of a rare variant does not demonstrate a causal relationship with the lethal event, while variants of uncertain significance should not be used to attribute a genetic cause to death in the absence of further evidence. The increasing availability of genomic data therefore makes a multidisciplinary interpretative process based on standardized criteria indispensable [15,16].

### 2.1. Definitions and Clinical Scope of Molecular Autopsy

A clear distinction among SCD, SUD, SADS, and autopsy-negative sudden death is essential for defining the appropriate clinical scope of MA. These terms should not be considered interchangeable [13].

SCD refers to an unexpected natural death attributable to a cardiac cause, which may result from structural heart disease, coronary artery disease, cardiomyopathy, myocarditis, aortic disease, primary electrical disorders, or other cardiac conditions. In contrast, sudden unexplained death refers to a sudden unexpected death in which a comprehensive investigation, including complete autopsy, histological examination and toxicological assessment, fails to identify a sufficient cause of death.

SADS is generally used to describe cases of SUD in which no structural or toxicological cause is identified, and the heart is morphologically normal or shows no sufficient pathological substrate to explain the death. This category is particularly relevant to inherited channelopathies and other primary electrical disorders but may also include early or incompletely penetrant forms of cardiomyopathy.

The distinction is clinically relevant because not every case of SCD represents an indication for MA. Post-mortem genetic testing is particularly informative in SUD/SADS, in young decedents with an unexplained or inconclusive autopsy, in cases showing a phenotype suggestive of inherited cardiovascular disease, and when the personal or family history raises suspicion of a hereditary condition.

Accordingly, the diagnostic pathway should be conceptualized as:

SCD → comprehensive post-mortem investigation → identified structural/toxicological cause or unexplained death → SUD/SADS evaluation → phenotype assessment → MA when clinically indicated.

MA should therefore be regarded as an integrated component of post-mortem investigation rather than as a genetic test applied indiscriminately to all sudden deaths.

Contemporary European recommendations further support the integration of genetic evaluation into the investigation of selected cases of sudden death. The 2022 ESC Guidelines on ventricular arrhythmias and the prevention of sudden cardiac death include specific recommendations for the diagnostic evaluation of sudden cardiac death victims and relatives of SADS decedents, as well as a dedicated framework for genetic testing [19]. Within this framework, genetic evaluation should be interpreted in conjunction with the clinical and phenotypic context and preferably performed within experienced multidisciplinary settings. Thus, molecular autopsy should no longer be regarded exclusively as a research procedure but as a component of an integrated diagnostic pathway in appropriately selected cases.

### 2.2. From Genetic Finding to Causal Inference

The identification of a genetic variant should not be considered synonymous with the identification of the cause of death. The clinically relevant question is not simply whether a genetic alteration is present, but whether the available evidence supports the conclusion that an inherited cardiovascular disorder contributed to the lethal event.

Causal inference requires the integration of multiple domains of evidence, including the post-mortem phenotype, histopathological findings, toxicological results, circumstances of death, personal and family history, gene–disease validity, variant-level evidence, segregation, functional data, penetrance, inheritance pattern, and genotype–phenotype concordance.

Accordingly, the interpretation of an MA result should follow a hierarchical diagnostic model:

Gene–disease validity → pathogenic mechanism → variant classification → phenotype concordance → familial segregation → penetrance and expressivity → causal inference.

A pathogenic or likely pathogenic variant may provide strong evidence of an inherited predisposition, but the final attribution of death should remain based on the integration of genetic and clinicopathological evidence. Conversely, a negative genetic result does not exclude an inherited disorder, because current testing strategies do not detect all possible pathogenic mechanisms.

**Figure 1:**
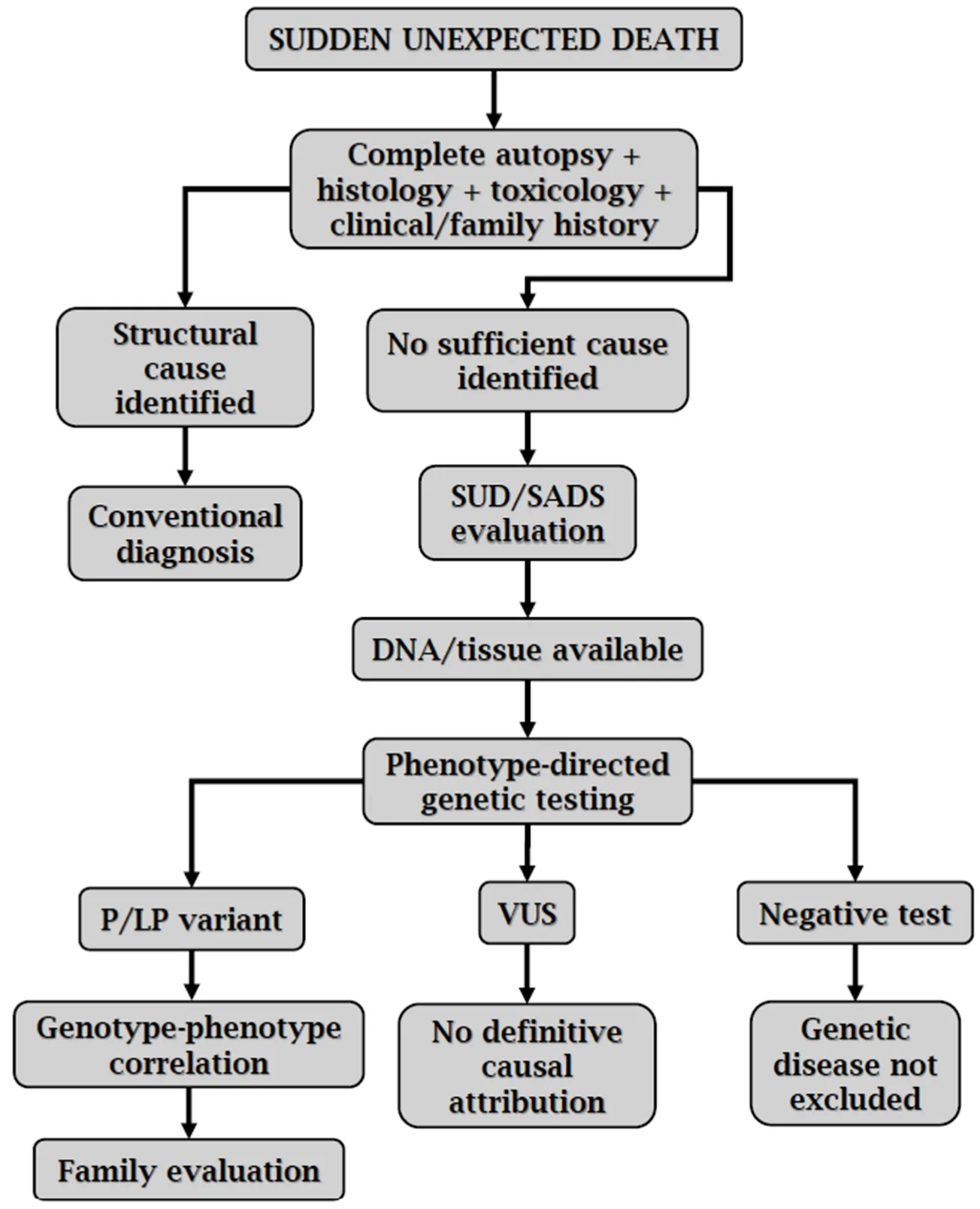
Clinical diagnostic pathway of molecular autopsy in sudden unexplained death.

Figure 1 illustrates the clinical diagnostic pathway of MA in SUD, integrating comprehensive post-mortem examination, histopathological and toxicological findings, clinical and family history, phenotype-directed genetic testing, variant interpretation, and subsequent evaluation of relatives when indicated.

### 2.3. Integration with Autopsy, Histopathology, and Toxicology

The diagnostic value of MA increases when genetic data are integrated with macroscopic autopsy, histopathological examination and toxicological investigation [19,20,21]. Autopsy remains the first line of investigation and allows for the identification or exclusion of major structural causes of SCD, including coronary artery disease, cardiomyopathies, myocarditis, valvular abnormalities, and aortic pathology. Histological examination subsequently allows us to characterize microscopic alterations potentially responsible for the event, such as fibrosis, myocardial necrosis, inflammatory infiltrates, or alterations in the cardiac structure. Therefore, genetics should be interpreted as a complementary component of the investigation and not as an alternative to morphological evaluation [14,17].

The importance of integrating molecular data and cardiovascular histopathology is particularly evident in early or phenotypically nuanced forms of cardiomyopathy. Findings such as myocardial fibrosis, alterations in ventricular structure or nonspecific cellular changes may acquire greater diagnostic significance when associated with a genetic variant consistent with the phenotype [17]. Conversely, the absence of a significant histological substrate may strengthen the hypothesis of a primary channelopathy, especially when a pathogenic variant in a gene associated with an inherited arrhythmic disorder is identified [14,15].

Forensic toxicology also plays an essential role in the interpretation of MA [19,20,21]. The presence of pharmacological or recreational substances should not be automatically considered as the cause of death but must be evaluated in the context of individual genetic predisposition. In a cohort of young victims of SCD, the integration of toxicological and molecular data highlighted that some substances, including drugs and caffeine, can act as potential triggers of arrhythmias in genetically predisposed subjects [19]. This concept is particularly relevant in channelopathies, in which environmental or pharmacological factors can precipitate an arrhythmic event in the presence of a pre-existing genetic substrate. Toxicology should therefore be interpreted not only in terms of drug concentration, but also considering the victim’s age, clinical history, context of death, autopsy findings, and genetic profile [19].

MA should be included in an integrated diagnostic pathway, in which anamnestic data, circumstances of death, macroscopic autopsy, histopathology, toxicology and genetics converge towards a unitary etiological evaluation. This multidisciplinary model is particularly important in complex cases, in which no single investigation can independently establish the cause of death [14,15,16]. The integrated approach also allows us to distinguish a genetically relevant variant from an incidental finding and to reduce the risk of inappropriate causal attributions. The most recent evidence therefore supports a model in which the pathologist, the geneticist, the cardiologist and the forensic doctor collaborate in the case selection phase, interpretation of the results and definition of the clinical implications for the family members [15,16].

MA becomes a diagnostic bridge between postmortem pathology and preventive cardiovascular medicine, allowing us to move from the simple classification of death as unexplained to the identification, when possible, of a genetic mechanism susceptible to being sought in family members. The correct integration of genetic data with morphological and toxicological evidence therefore constitutes the fundamental prerequisite for the post-mortem result to acquire a real diagnostic and clinical significance [8,14,16].

The main clinical and forensic indications for molecular autopsy, together with its integration with conventional post-mortem investigations, are summarized in Table 1.

## 3. Genetic Causes of Sudden Cardiac Death

Inherited cardiovascular diseases are a major cause of SCDY and include a broad spectrum of conditions characterized by structural alterations of the myocardium, abnormalities of the cardiac electrical activity, or a combination of both. The genetic pattern of inherited SCD has traditionally been attributed to monogenic diseases with variable penetrance. However, more recent evidence suggests a more complex genetic architecture, in which multiple variants, genetic modifiers and environmental factors may contribute to phenotypic manifestation and individual arrhythmic risk [22,23]. The distinction between hereditary cardiomyopathies and channelopathies remains particularly relevant, since the former may present variable macroscopic and histopathological alterations, while the latter may determine malignant arrhythmia in the presence of an apparently normal heart [22,24].

### 3.1. Hereditary Cardiomyopathies

Hereditary cardiomyopathies mainly include hypertrophic cardiomyopathy (HCM), dilated cardiomyopathy (DCM), arrhythmogenic cardiomyopathy (ACM) and, in specific clinical-genetic contexts, non-compaction cardiomyopathy and other overlapping phenotypic forms [22,25]. These conditions show marked genetic and phenotypic heterogeneity and may manifest with heart failure, ventricular arrhythmias or SCD as the first clinical manifestation [22,25,26].

HCM is one of the main inherited cardiomyopathies associated with SCD and is predominantly related to variants in genes encoding sarcomere proteins. Among the most implicated genes are *MYBPC3*, *MYH7*, *TNNT2*, and *TNNI3*, while additional genes may contribute to rarer phenotypes or genetically complex forms. In MA, the identification of a pathogenic variant in a sarcomeric gene may be particularly relevant in subjects with ventricular hypertrophy, myocyte disorganization, or fibrosis, but also in cases where the structural phenotype is minimal or incompletely expressed. Recent recommendations therefore emphasize the importance of correlating the genetic finding with the anatomopathological phenotype, avoiding considering identified rare variants as causative [25,26].

DCM presents a high genetic heterogeneity and includes forms associated with variants in genes involved in the structure of the sarcomere, the nuclear envelope, the cytoskeleton, and cell adhesion complexes. Among the most relevant genes are *TTN* and *LMNA*, along with other genes, including *FLNC*, *DSP*, *RBM20*, and *DES*, associated with phenotypes characterized by a significant arrhythmogenic component. The presence of a pathogenic variant may therefore be particularly important in cases of DCM associated with ventricular arrhythmias, conduction disturbances, or a family history of SCD [25,26].

ACM represents a particularly relevant group in the context of juvenile SCD and exercise-related events. Classic forms are frequently associated with variants in desmosomal genes, such as *PKP2*, *DSP*, *DSG2*, and *DSC2*, while variants in non-desmosomal genes, including *FLNC* and *TMEM43*, can determine complex arrhythmogenic phenotypes. Recent data from a large cohort of athletes subjected to molecular autopsy have identified clinically actionable variants, with a particular contribution from genes associated with cardiomyopathies, including *FLNC* and *TMEM43* [7]. These findings underscore how exercise-related SCD may represent the first manifestation of a genetically determined cardiomyopathy even in the absence of a previously recognized clinical phenotype [25,26].

A further element of complexity is the possible overlap between genetic predisposition and acquired factors. Recent autopsy studies have highlighted that viral myocarditis, and genetically determined cardiomyopathy can coexist and contribute synergistically to lethal events. This observation is particularly important for MA, since a myocardial inflammatory finding does not necessarily exclude an underlying genetic predisposition. On the contrary, the presence of a pathogenic variant may provide an interpretative key to understanding why an apparently limited inflammatory process can be associated with a fatal arrhythmic event [27].

### 3.2. Channelopathies

Cardiac channelopathies are inherited electrical disorders characterized by abnormal function of cardiac ion channels or proteins involved in regulating myocardial excitability. Unlike cardiomyopathies, they may manifest in the absence of significant structural alterations, making conventional autopsy potentially negative. Among the main channelopathies associated with SCD are long QT syndrome (LQTS), Brugada syndrome (BrS), short QT syndrome (SQTS), catecholaminergic polymorphic ventricular tachycardia (CPVT), and, in some cases, idiopathic ventricular fibrillation and early repolarization syndromes [11,14,28,29].

LQTS is characterized by prolonged ventricular repolarization and an increased risk of torsades de pointes, ventricular fibrillation, and SCD. The most genetically established forms are mainly associated with *KCNQ1*, *KCNH2*, and *SCN5A*, while other genes contribute to rarer forms [24,28,29]. BrS, on the other hand, is characterized by a predominantly electrical arrhythmogenic substrate and a characteristic electrocardiographic pattern, with *SCN5A* being the main associated gene in genetically defined cases [24,28,29]. *SQTS* is a rare channelopathy characterized by a pathologically short QT interval and a high risk of atrial fibrillation and ventricular arrhythmias. The main implicated genes are *KCNH2*, *KCNQ1*, *KCNJ2*, *CACNA1C*, *CACNB2*, and *CACNA2D1* [24,28,29]. However, the relationship between individual variants and clinical phenotype can be complex, and not all variants identified in channelopathy-associated genes have sufficient evidence to be considered causative.

CPVT is another important cause of SCDY, generally associated with variants in genes involved in intracellular calcium regulation, particularly *RYR2* and, more rarely, *CASQ2*. The disease can present with ventricular arrhythmias induced by exercise or adrenergic stress in the absence of significant cardiac structural abnormalities. For this reason, in cases of unexplained SCD, the identification of a pathogenic variant in *RYR2* can provide important etiological evidence and allow for a targeted evaluation of family members [24,28,29].

Channelopathies therefore constitute a particularly relevant paradigm for MA, as a morphologically normal heart does not necessarily equate to a genetically normal heart. Recent reviews also emphasize that the genetics of channelopathies is increasingly complex, with growing attention to incomplete penetrance, genetic modifiers, and the possible interaction between multiple variants and environmental factors [24,28,29]. This makes rigorous interpretation of the variants and careful correlation with the circumstances of death and available clinical data essential.

### 3.3. Other Genetic Conditions

In addition to cardiomyopathies and channelopathies, the increasing availability of genomic technologies has expanded the number of genetic conditions potentially associated with SCD. Among these hereditary aortopathies, some phenocopies of cardiomyopathy and multisystem conditions in which cardiac involvement may represent the first clinical manifestation are of relevance [22]. Variants associated with inherited aortic syndromes can lead to aortic dissection or rupture as a sudden and fatal event, while some multisystem genetic conditions may present with initially subtle cardiac phenotypes [30].

Another emerging area is represented by variants in genes traditionally not classified as major SCD genes but progressively associated with arrhythmogenic or cardiomyopathic phenotypes.

Indeed, the increasing identification of new genes and rare variants raises a fundamental question: expanding the number of genes analyzed does not necessarily equate to a proportional increase in diagnostic certainty. The use of increasingly larger panels increases the probability of identifying VUS or variants in genes for which the relationship with SCD is not yet sufficiently consolidated [22]. It is therefore necessary to distinguish genes with a well-documented causal association from those that are emerging or have limited evidence, avoiding attributing an etiological significance to genetic results that are not adequately supported [24].

The increasing availability of genomic data also suggests a progressive shift from an exclusively monogenic model to a more complex view of SCD predisposition. The most recent evidence indicates that, at least in some conditions, arrhythmic risk can be influenced by the combination of rare and common variants, genetic modifiers and environmental factors [22]. This perspective could change the interpretation of MA in the future, shifting attention from the simple search for a single causative variant towards an integrated evaluation of individual genetic predisposition.

Overall, the current genetic spectrum of SCD thus encompasses a continuum from structural cardiomyopathies to electrical channelopathies and emerging genetic conditions with overlapping phenotypes. Knowledge of this spectrum is essential to define appropriate MA strategies and to interpret the genetic result in the correct clinicopathological context. Characterization of the variant, however, must always be accompanied by the evaluation of the victim’s phenotype and, when possible, by clinical and genetic analysis of family members, to distinguish a real hereditary predisposition from an incidental genetic finding.

The major inherited cardiac conditions associated with SCD, their representative genes, pathological phenotypes, and relevance to MA are summarized in Table 2.

## 4. Advances in Postmortem Genetic Testing

The evolution of sequencing technologies has profoundly changed the approach to MA, allowing the transition from the targeted search for single variants to the simultaneous analysis of numerous genes and, more recently, of the entire exome or genome. The current development of post-mortem genomics is oriented not only to increase the diagnostic yield, but also to define more selective, reproducible and autopsy phenotype-adapted analysis strategies [31,32,33]. The choice of platform and analytical approach must therefore be considered an integral part of the diagnostic process and must consider the quality of the available biological material, the clinical-pathological suspicion and the need to limit the identification of VUS [31,32,33].

### 4.1. Targeted Gene Panels

Targeted gene panels allow the simultaneous sequencing of a predefined set of genes associated with cardiomyopathies, channelopathies, and other inherited conditions responsible for SCD. The main advantage is represented by the high sequencing depth, the relative speed of the analysis and the possibility of concentrating the interpretation on genes with a consolidated gene–disease association [4,31,32]. A targeted approach is particularly appropriate when autopsy findings point towards a specific phenotype, for example a hypertrophic, dilated or arrhythmogenic cardiomyopathy, or a channelopathy [4,31].

In 2024, Del Duca et al. proposed a first- and second-line panel model specifically targeting post-mortem SCD, distinguishing the genes most relevant for HCM, DCM, ACM/ARVC and channelopathies from those to be analyzed subsequently in selected cases [4]. This strategy represents an important attempt to standardize MA, since it prevents the indiscriminate expansion of the number of genes analyzed from simply increasing the number of VUS without a corresponding increase in diagnostic certainty. Recent studies in the cardiovascular field have also demonstrated that the use of larger panels can increase the diagnostic yield, but at the same time determines an increase in VUS and incidental findings, making rigorous interpretation according to shared criteria indispensable [34].

Targeted panels do have some limitations, however. The analysis is necessarily conditioned by preliminary knowledge of the genes associated with the disease and may therefore not identify variants located in emerging genes or genes not yet included in the panel. Furthermore, some structural variants, deep intronic alterations, genomic expansions or complex rearrangements may escape conventional short-read sequencing-based methods [5,25,32]. For these reasons, in negative cases or those characterized by a particularly complex phenotype, the use of more extensive genomic methods can offer additional diagnostic value.

### 4.2. Whole-Exome Sequencing and Whole-Genome Sequencing

WES allows for the simultaneous analysis of most coding regions of the genome and is an important alternative or addition to targeted panels. Its main advantage is the possibility to identify variants in genes not initially considered based on phenotype, thus allowing a more exploratory approach to SCD [32]. An Italian study published in 2024 on subjects with juvenile SCD demonstrated the potential of WES associated with a personalized virtual gene panel: the extended approach identified potentially causative variants in a higher percentage than the analysis limited to conventional cardiac panels, with a particular advantage in subjects with structurally normal heart [17].

These results suggest that WES may be particularly useful in autopsy-negative or phenotypically complex cases, in which a restricted panel may not identify the genetic cause. Exome analysis also allows for subsequent re-evaluation of data using new virtual panels, without the need to repeat sequencing if new genes or new gene–disease associations are identified [17].

WGS is a further advancement, as it theoretically allows interrogation of both coding and non-coding regions of the genome. Compared to WES, WGS can offer better ability to identify structural variants, deep intronic variants, regulatory alterations and other genomic modifications that are difficult to detect by targeted approaches [5,32]. However, the greater volume of data generated by WGS also leads to greater bioinformatic and interpretative complexity. In the context of SCD, therefore, WGS should not be considered simply as a broader version of the gene panel, but as a strategy that requires bioinformatics infrastructure, up-to-date databases and specific expertise for variant classification [32].

A particularly important aspect is the relationship between the breadth of the sequencing and the clinical significance of the result. Increasing the number of genes analyzed can increase the possibility of identifying rare variants, but also the probability of obtaining incidental or VUS findings. This principle is particularly relevant in MA, where the absence of a clinical phenotype in the victim can make it even more difficult to establish the pathogenicity of a variant.

Importantly, broader sequencing should not be considered synonymous with a better MA. The diagnostic value of WES or WGS depends on the pre-test probability of an inherited disorder, the availability of an interpretable phenotype, the strength of gene–disease associations, and the expertise of the diagnostic laboratory.

In phenotype-negative SUD, hypothesis-free WES/WGS may substantially increase the burden of VUS, incidental findings, and variants in genes with limited or disputed disease associations. Accordingly, broad sequencing should be preferentially considered in unresolved or particularly complex cases within experienced multidisciplinary or research settings, whereas phenotype-directed testing remains preferable when a specific inherited cardiovascular disorder is suspected.

The choice of sequencing strategy should therefore balance diagnostic breadth against interpretative specificity rather than simply maximizing the number of genes analyzed.

### 4.3. Long-Read Sequencing and New Technologies

Long-read sequencing technologies are one of the most exciting frontiers in genomics applied to hereditary diseases. Unlike conventional short-read platforms, long-read sequencing allows for the analysis of much larger DNA fragments, facilitating the identification of structural variants, large insertions and deletions, repetitive expansions, and complex genomic regions [22]. These features could be particularly relevant in cardiomyopathies and genetic syndromes in which a portion of the pathogenetic variability is not easily identifiable by conventional sequencing.

Interest in long-read sequencing also stems from the possibility of simultaneously characterizing sequence variants and genomic structure and, in certain platforms, of obtaining additional information on genome organization. Although the use of SCD in MA is still in a predominantly emerging phase compared to targeted panels and WES/WGS, this technology could contribute in the future to the diagnosis of cases previously classified as genetically negative [22]. Its use, however, will require further clinical validation, standardization of protocols, and the availability of high-quality and quantitative DNA.

Furthermore, the integration of genomics with multi-omics approaches, including transcriptomics, epigenomics, proteomics and functional analyses, could help to clarify the biological significance of variants not immediately classifiable through sequence data alone and to improve the distinction between pathogenic variants and incidental findings [22,32]. In SCD, this could be particularly useful in cases where a rare variant is identified but not sufficiently characterized by current databases.

The main post-mortem sequencing approaches currently available, including their advantages, limitations, and potential applications in sudden cardiac death, are summarized in Table 3.

### 4.4. Biological Samples and Pre-Analytical Issues

The pre-analytical phase is one of the most critical aspects, as the quality and type of biological sample directly influence the possibility of obtaining DNA suitable for sequencing. Ideally, material intended for genetic analysis should be collected early during autopsy, before prolonged exposure to potentially degrading environmental conditions, and stored according to standardized protocols [31,32]. Peripheral blood is generally the preferred sample when available, while fresh or frozen tissues, particularly myocardium, spleen, and skeletal muscle, can also be used.

The availability of adequate samples is particularly important because, in many forensic cases, biological material is collected exclusively for histological and toxicological purposes, and only subsequently is the indication for AM identified. In such circumstances, formalin-fixed, paraffin-embedded (FFPE) tissues may be predominantly available, as DNA fragmentation and modifications can compromise the quality of sequencing [35]. DNA quality can be further compromised by the postmortem interval, temperature, the presence of autolytic phenomena, tissue type, and preservation methods [33]. When victim material is insufficient or inadequately preserved, family DNA can represent an important indirect diagnostic resource [10,36].

Sample management should therefore be considered an integral part of the autopsy protocol, and not a post-anatomy phase. Early collection of fresh or frozen blood and/or tissue, correct sample identification and traceability, appropriate preservation, and documentation of pre-analytical conditions can determine the very possibility of performing a reliable MA [31,33]. This aspect takes on relevance in systems in which forensic autopsy and genetic analysis are performed in different facilities, requiring close collaboration between the pathologist, forensic doctor, genetics laboratory, and clinical geneticist.

Looking ahead, the evolution of molecular autopsy will therefore not only depend on the availability of increasingly powerful sequencing platforms, but also on the ability to standardize the entire diagnostic process, from sample collection to variant classification. An integrated approach combining targeted panels, WES/WGS, and, in selected cases, long read and multi-omics technologies could progressively reduce the proportion of genetically unresolved SCDs, while maintaining high levels of diagnostic accuracy [10,31,32,33,36,37].

## 5. Genetic Variant Interpretation

Interpreting genetic variants is one of the most complex steps in MA, as identifying a rare variant does not necessarily equate to identifying the genetic cause of death. The increase in sequencing capacity has in fact determined a considerable increase in the number of variants detected per individual, making their correct classification and the definition of their biological and clinical relevance a central element of the entire diagnostic process. In SCD, this issue is particularly relevant because the victim’s phenotype may be incomplete, absent, or no longer directly assessable and because a variant identified post-mortem must be interpreted in the context of autopsy findings, family history, and, when available, family members’ clinical data [4,11].

Importantly, negative or non-diagnostic molecular results remain common. The absence of a pathogenic or likely pathogenic variant does not exclude a genetic contribution to SCD, particularly in disorders with incomplete knowledge of the genetic substrate, incomplete penetrance, or variants not detectable by the selected sequencing strategy. Conversely, identification of a rare variant does not necessarily provide additional diagnostic information unless the variant has sufficient evidence of pathogenicity and is compatible with the post-mortem phenotype. Therefore, the clinical utility of MA should be assessed not simply according to the proportion of genetic findings, but according to the proportion of cases in which the result provides a clinically and biologically interpretable explanation of death or actionable information for relatives.

### 5.1. Variant Classification

Variant classification in MA is commonly based on the ACMG/AMP framework, which integrates different categories of evidence including population frequency, functional data, segregation, clinical observations, computational evidence, and variant characteristics [38,39]. However, the generic ACMG/AMP framework should not be considered sufficient in isolation for cardiovascular genetic interpretation [40,41].

For inherited cardiovascular diseases, disease- and gene-specific specifications developed or endorsed by expert groups, particularly within the ClinGen framework, provide additional guidance for the application and weighting of individual ACMG/AMP criteria. These specifications are particularly relevant when the pathogenic mechanism, penetrance, phenotype specificity, or expected consequence of a variant is gene dependent [40].

A further prerequisite is the assessment of gene–disease validity. The classification of a variant should therefore not begin with the molecular alteration alone, but with the strength and specificity of the association between the gene and the disease phenotype. This distinction is particularly important in molecular autopsy, where broad sequencing may identify variants in genes with limited, disputed, or insufficient evidence for disease causation [42].

Thus, a contemporary interpretative framework (Table 4) can be summarized as:

Gene–disease validity → disease mechanism → variant-level evidence → phenotype concordance → clinical/familial evidence → causal inference.

Not all loss-of-function variants are necessarily pathogenic in all genes, and the molecular consequence of a variant must always be interpreted in relation to the established disease mechanism of the specific gene.

### 5.2. Pathogenic Variants and Variants of Uncertain Significance

The distinction between pathogenic/probably pathogenic (P/LP) variants VUS is fundamental. A variant classified as pathogenic or probably pathogenic, when consistent with the phenotype and disease mechanism, may provide important evidence in favor of a genetic predisposition to SCD. Conversely, a VUS should not be used as sufficient evidence to attribute a genetic cause to death nor to make definitive clinical decisions in family members [38,39].
**Key clinical principle**A VUS should not be used as evidence that a genetic variant caused sudden death and should not be used as a predictive genetic test to determine the clinical management of unaffected relatives. VUS findings require continued interpretation in the context of phenotype, family data, gene–disease validity, functional evidence, and evolving genomic knowledge.

The presence of VUS represents one of the main limitations of MA. The widespread use of NGS and WES has increased the ability to identify rare variants but has simultaneously increased the number of findings of uncertain significance, so that the mere presence of a rare variant should not be interpreted as evidence of causality [43].

The classification of genetic variants is dynamic and should therefore be periodically reassessed. Fernandez-Falgueras et al. [44] reinterpreted 1425 variants identified in 618 individuals evaluated for inherited cardiovascular diseases and SCD and reported an overall reclassification rate of 36.21%. However, the direction and clinical significance of reclassification were highly heterogeneous. Most reclassifications resulted in a reduction in clinical impact, whereas only 3.93% of variants were reclassified toward higher-impact categories. Among variants initially classified as VUS, 25.86% were downgraded to likely benign/benign categories, whereas only 2.64% were upgraded to likely pathogenic/pathogenic categories.

These findings emphasize that a high overall reclassification rate should not be interpreted as evidence that a comparable proportion of variants becomes clinically actionable. Rather, they support the need for periodic reinterpretation, particularly in view of evolving gene–disease knowledge, population databases, segregation data and functional evidence [44,45].

### 5.3. Genotype–Phenotype Correlation

Genotype–phenotype correlation is a crucial step in determining whether an identified variant is associated with the lethal event. In MA, the genotype should be compared with macroscopic and microscopic findings, clinical history, electrocardiogram if available, circumstances of death and family history [37,38].

This correlation is particularly important in cardiomyopathies. A pathogenic variant in a sarcomeric gene, for example, becomes more relevant when it is associated with myocardial hypertrophy, disarray, fibrosis, or other findings consistent with hypertrophic cardiomyopathy. Similarly, a variant in a desmosomal gene may assume greater significance when associated with morphological and histopathological features compatible with arrhythmogenic cardiomyopathy. Conversely, the complete absence of a compatible phenotype should induce caution in the causal attribution of the variant.

Phenotypic correlation can, however, be difficult in individuals who die suddenly, because some inherited diseases have incomplete penetrance and may not have yet produced recognizable structural alterations at the time of death. This is particularly evident in channelopathies, in which the molecular substrate can determine a predominantly electrical phenotype without significant macroscopic or histological abnormalities [38,43]. In these cases, the absence of morphological alterations should not be considered as evidence against the relevance of the variant.

A particularly useful approach is the analysis of family members. Segregation of the variant with the phenotype within the family can provide important evidence for its classification, while identification of the same variant in clinically affected or unaffected family members can help define penetrance and expressivity [37,38]. In the postmortem setting, therefore, genetic investigation of the victim can constitute the starting point of a broader family diagnostic process.

### 5.4. Penetrance, Expressivity, and Genetic Modifiers

The presence of a pathogenic variant does not necessarily determine the clinical expression of the disease in all carriers. This phenomenon is particularly important in cardiomyopathies and channelopathies, in which individuals carrying the same variant may present different phenotypes or remain clinically asymptomatic throughout their lives [43,46].

Variable expressivity represents a related phenomenon, in which the same variant can cause clinical manifestations of varying severity. Therefore, within the same family, asymptomatic subjects, individuals with a subclinical phenotype and patients with severe arrhythmias or SCD may coexist. This variability makes it particularly risky to infer the penetrance of a variant solely from the victim’s phenotype.

The most recent evidence also indicates that penetrance and disease severity can be influenced by genetic modifiers, common variants, and non-genetic factors. Hereditary cardiomyopathies should therefore not necessarily be considered exclusively monogenic conditions, the final phenotype may result from the interaction between a main variant, the individual genetic background and environmental or behavioural factors [46].

This perspective is particularly relevant for SCD, as it suggests that arrhythmic risk may arise not exclusively from the presence of a single pathogenic variant, but from a combination of genetic and non-genetic factors [43]. Although these mechanisms require further validation, they represent a possible explanation for the marked phenotypic variability observed in carriers of the same variant.

The interpretation of a variant identified during a molecular autopsy should therefore follow a hierarchical model: gene validity → pathogenic mechanism → variant classification → genotype–phenotype correlation → familial segregation → evaluation of penetrance and modifiers. This approach allows us to reduce the risk of mistakenly attributing death to an incidental genetic finding and allows us to transform the molecular data into information that is truly useful for the victim and, above all, for his family [37,38,41].

## 6. Clinical, Forensic and Ethical Implications

MA does not end with the identification of a genetic variant in the deceased but can represent the starting point of a diagnostic and preventive approach aimed at the family. The identification of a pathogenic or probably pathogenic variant associated with a cardiomyopathy or a channelopathy can in fact allow for the early recognition of carrier family members and the initiation of personalized cardiac and genetic surveillance programs. The most recent evidence highlights how the integration between post-mortem investigation, genetics and family evaluation can transform the MA from an exclusively forensic diagnostic tool to a tool for the prevention of sudden cardiac death in subsequent generations [3,47,48].

### 6.1. Cascade Screening and Clinical Evaluation of Relatives

Cascade evaluation of relatives is one of the most clinically important consequences of a molecular autopsy that identifies a well-established pathogenic or likely pathogenic variant associated with an inherited cardiovascular disorder [47,49].

The molecular result, however, represents only the starting point of the familial diagnostic process. At-risk relatives may require genetic counseling, targeted genetic testing, electrocardiography, echocardiography, exercise testing, ambulatory monitoring, cardiac magnetic resonance imaging, provocative testing, or longitudinal clinical surveillance according to the suspected disease and individual phenotype.

When a pathogenic or likely pathogenic familial variant is identified, cascade genetic testing can help distinguish relatives who carry the familial variant from those who do not. However, genetic status should be interpreted together with clinical phenotype, age, penetrance, and variable expressivity [50,51].

Importantly, the absence of an identified pathogenic variant in the deceased does not necessarily eliminate the need for clinical assessment of first-degree relatives. In young SUD/SADS cases, family evaluation may remain appropriate even when molecular autopsy is negative or non-diagnostic. Clinical assessment should therefore not be regarded as subordinate to molecular testing, but as an independent component of familial risk evaluation [52].

Accordingly, the preventive value of MA lies not simply in identifying genetic carriers, but in establishing an integrated pathway involving genetic counseling, phenotype-directed clinical screening, cascade testing when appropriate, and longitudinal follow-up [13].

The potential MA findings and their implications for relatives, including genetic counselling, cascade screening, and clinical surveillance, are summarized in Table 5.

### 6.2. Medico-Legal and Ethical Implications

MA lies at the intersection of forensic medicine, clinical genetics, pathological anatomy, and cardiovascular prevention, generating unique ethical and medicolegal issues.

From a medicolegal perspective, the integration of conventional autopsy and genetic analysis can increase the accuracy of determining the cause of death in cases of unexplained SCD. Correct interpretation of genetic data can therefore serve a dual function, clarify the victim’s death and prevent further events in the family [10,50].

The ethical dimension is particularly complex because the subject being tested is deceased and cannot directly express his or her will. The outcome may, however, have important consequences for living family members, creating a tension between the protection of the deceased’s privacy and the possible right of family members to be informed of a preventable health risk [53]. Managing this situation requires a clear regulatory framework and the involvement of professionals adequately trained in clinical genetics and counseling.

A further issue concerns the possibility that the genetic result may be interpreted outside of its proper clinical context. Identifying an insufficiently characterized variant, especially a VUS, can lead to anxiety, overmedicalization, or unnecessary diagnostic procedures in family members. Therefore, the communication of the result must be accompanied by an explanation of the degree of diagnostic certainty and the limitations of the analysis [38,48].

Recent multidisciplinary experiences therefore underline the need to define shared protocols that establish who can request post-mortem testing, which samples must be stored, how the results must be classified and according to which methods the relatives can be informed [50]. The lack of uniform procedures still represents one of the obstacles to the full integration of MA into medico-legal practice [48,50].

### 6.3. Privacy and Management of Genetic Results

Data management must simultaneously guarantee confidentiality, security, traceability, and the possibility of clinical use when the result has preventive implications [48,53].

Specifically, data management should include a clear distinction between raw genetic data, diagnostic interpretation, and clinically communicable result. Sequencing can, in fact, produce numerous variants with no immediate clinical significance, and indiscriminately sharing all this information with family members can lead to misinterpretations. Instead, sufficiently validated and clinically relevant results should be communicated as a priority, through a genetic counselling process [38,48].

A further issue concerns the long-term preservation of genomic data. Evolving knowledge may allow the reinterpretation of variants initially classified as VUS and transform a previously uninformative result into a clinically relevant finding. Data retention must therefore be sufficiently structured to allow, when appropriate, subsequent re-evaluation [38,48].

Communicating results to family members also requires a delicate balance between respecting privacy and the possibility of preventing a potentially life-threatening event. Genetic information of the victim should not be disclosed indiscriminately, but its relevance for family members at risk makes it necessary to define procedures that allow, in compliance with the applicable legal framework, to reach the subjects potentially benefiting from the information [50,53].

Genetic counseling is therefore essential. It should, when possible, preceded testing of family members and accompany the communication of the results, explaining their meaning, limitations, probability of transmission, the possibility of disease expression, and implications for other family members. This approach is particularly important in cases where the result could determine significant changes in cardiac surveillance or therapeutic choices.

The protection of privacy, the correct interpretation of variants, and the definition of professional and medico-legal responsibilities represent indispensable conditions for an ethically sustainable application of post-mortem genomics.

## 7. Current Challenges and Future Perspectives

Despite the rapid development of NGS and postmortem genetic analysis methods, MA still presents numerous diagnostic, methodological and interpretative criticalities. A significant proportion of SCD cases remain genetically unresolved, which may reflect limitations of current sequencing methods, the presence of variants located in poorly characterized regulatory or non-coding regions, structural variants, genes not yet associated with inherited heart disease, or pathogenic mechanisms not yet fully understood [16,33,54]. Therefore, a negative result should not necessarily be interpreted as evidence of the absence of a genetic component of SCD, but rather as an expression of the current limitations of genomic knowledge and technologies.

Another important critical issue concerns the interpretation of genetic results. The increasingly widespread use of broad-spectrum sequencing strategies has led to a significant increase in the identification of rare variants, many of which cannot yet be classified with sufficient certainty. VUS remain frequent and their presence should not be considered indicative of causality in the absence of adequate clinical, pathological, functional or familial evidence [4,32]. The interpretation of postmortem variants is further complicated by incomplete penetrance and variable expressivity, especially in inherited cardiomyopathies and channelopathies. Therefore, genetic results should always be interpreted within an integrated framework that considers the circumstances of death, autopsy findings, histopathology, toxicology, available clinical information, and family history. This multidisciplinary approach is essential to distinguish a truly pathogenic finding from an incidental genetic variant.

Standardization is another priority. Intercenter differences in case selection, sampling procedures, tissue preservation, sequencing strategies, and variant classification may influence diagnostic yield and limit the comparability of published studies [4,33]. A standardized pathway should encompass the entire diagnostic process, from cardiovascular autopsy and appropriate specimen collection to molecular analysis, variant interpretation, and communication of clinically relevant results to family members. This model requires close collaboration between cardiac pathologists, forensic medicine specialists, cardiologists, geneticists and molecular genetics laboratories [4,32]. The definition of internationally shared protocols would also allow for improved quality control and a more reliable evaluation of the diagnostic performance of different molecular strategies.

Future perspectives could extend MA beyond simple DNA sequencing towards a more comprehensive multi-omics characterization. Integrating genomic data with transcriptomics, proteomics, epigenomics and other functional approaches could provide further evidence on the biological effects of rare or unclear variants and improve understanding of the molecular mechanisms underlying apparently unexplained SCD [55,56,57].This approach could be particularly useful when a potentially relevant variant is identified, but the available evidence is insufficient to establish its pathogenicity. However, post-mortem tissue degradation, the availability of adequately preserved biological material and the lack of standardized reference datasets are currently important limitations to the routine application of multi-omics in forensic and cardiovascular pathology [55,56,57].

AI also represents an emerging perspective for integrating the increasingly complex data generated by MA. Machine learning approaches could facilitate the prioritization of genetic variants and the integration of genomic, pathological and clinical information, potentially identifying patterns that are difficult to recognize using conventional analytical approaches [58,59]. AI could also contribute to the quantitative analysis of histopathological images and, in the future, to the integrated assessment of the genotype–phenotype relationship. However, these applications are still in their infancy in the post-mortem context and require large, properly annotated datasets, external validation, model transparency and evaluation of potential biases before they can be integrated into diagnostic practice [60,61].

Finally, international data sharing will be crucial to overcome the limited number of cases and the rarity of many potentially pathogenic variants. The integration of genomic, phenotypic and post-mortem data from multiple centers could improve variant classification, facilitate the identification of novel gene–disease associations and increase diagnostic yield. Interoperable databases that integrate clinical history, circumstances of death, autopsy and histopathological findings, genetic variants, and familial phenotypes could be particularly valuable for SCD research. In parallel, large-scale sharing of genomic data faces significant challenges related to privacy, consent, governance and secure management of genetic information [62,63].

The future of MA will not simply depend on the ability to sequence more genes, but on the ability to integrate increasingly diverse sources of information into a coherent diagnostic model. The combination of standardization of post-mortem investigations, advanced genomic technologies, multi-omics, AI and international data sharing could progressively transform MA from a complementary investigation in cases of unexplained SCD to an integrated precision medicine approach, capable not only of improving the definition of the cause of death, but also of identifying family members at risk and allowing targeted prevention strategies.

The major current challenges and emerging opportunities for the development and implementation of MA are summarized in Table 6.

## 8. Conclusions

SCD, especially in young people, remains a complex diagnostic challenge at autopsy, clinically, and genetically. In cases of negative autopsy, MA represents a fundamental extension of postmortem investigation, allowing the identification of genetic predispositions responsible for hereditary cardiomyopathies and channelopathies not necessarily associated with evident morphological alterations. In recent years, advances in sequencing technologies have significantly expanded diagnostic capabilities, allowing the analysis of a growing number of genes and the identification of variants previously undetectable using conventional methods.

The main limitation, however, is not the ability to identify variants, but the ability to establish their real biological and clinical significance. The increase in the amount of genomic data has in fact led to an increase in rare variants and VUS, making rigorous interpretation according to standardized criteria and a close correlation between genotype, phenotype, autopsy findings and family history essential. The presence of a rare variant should therefore not automatically be considered equivalent to the cause of death. This principle is particularly important in MA, where the risk of overinterpretation of a genetic finding can have not only diagnostic, but also clinical and familial consequences.

Furthermore, in a family setting, identifying a pathogenic variant in the victim can allow for the identification of asymptomatic carriers among family members and the initiation of a cascade screening, cardiac evaluation, and genetic counseling process. MA thus takes on a significance that goes beyond the definition of the disease and can transform a sudden and seemingly inexplicable event into an opportunity to prevent SCD in subsequent generations. Recent evidence confirms that clinical and genetic evaluation of family members can allow the identification of previously undiagnosed hereditary conditions and the adoption of personalized preventive strategies.

Therefore, standardization of autopsy protocols, sampling, tissue preservation, genetic analysis, and interpretation of variants is essential. The current heterogeneity between centers and countries limits the comparability of results and can impact the diagnostic yield of MA. A shared model should involve close integration between the cardiovascular pathologist, medical examiner, cardiologist, clinical geneticist, and genetics laboratory, from the victim’s examination to communicating the results to the family.

The integration of genomics with transcriptomics, proteomics, epigenomics, and other multi-omics strategies, along with the use of artificial intelligence, could enable more effective interpretation of rare variants, identify new pathogenic mechanisms, and integrate molecular, morphological, and clinical data. In parallel, the international sharing of genomic and phenotypic data through databases will be essential to improve the classification of variants and increase the ability to recognize new gene–disease associations.

An integrated workflow illustrating the transition from sudden cardiac death investigation to molecular diagnosis and familial prevention is presented in Table 7.

MA should be considered not as a simple postmortem genetic test, but as a multidisciplinary and translational diagnostic pathway that links pathology, forensic medicine, cardiology, genetics, and cardiovascular prevention, thus transforming a seemingly inexplicable death into a possible etiological diagnosis and, above all, an opportunity to identify and prevent at-risk family members.

## Figures and Tables

**Table 1:** Molecular autopsy: indications, workflow and complementary investigations.

Clinical/Forensic Scenario	Role of Molecular Autopsy	Complementary Investigations	Expected Outcome
SCD in a young individual with negative or inconclusive autopsy	Identification of an inherited arrhythmic or cardiomyopathic substrate	Complete cardiovascular autopsy, histopathology, toxicology	Identification of a potential genetic cause
SCD with structurally normal heart	Investigation of channelopathies and other primary electrical disorders	ECG history, family history, toxicology	Molecular diagnosis of an inherited arrhythmic syndrome
SCD with findings suggestive of cardiomyopathy	Identification of pathogenic variants associated with inherited cardiomyopathies	Histopathology, cardiac imaging when available, family history	Genotype–phenotype correlation
SCD with a positive family history	Identification of a familial pathogenic variant	Three-generation pedigree, clinical screening of relatives	Identification of relatives at increased risk
Unexplained SCD after conventional autopsy	Investigation of genetic causes not detectable by morphological examination	Histology, toxicology, molecular testing	Etiological diagnosis or genetic risk stratification
Suspected inherited cardiac disease with equivocal phenotype	Detection and interpretation of potentially disease-associated variants	Detailed pathological and clinical assessment	Integrated molecular and phenotypic diagnosis

**Table 2:** Major genetic causes of sudden cardiac death.

Disease Category	Representative Genes	Main Phenotype	Typical Pathological Findings	Role of Molecular Autopsy
Hypertrophic cardiomyopathy	*MYH7*, *MYBPC3*, *TNNT2*, *TNNI3*	Ventricular arrhythmias, SCD	Myocardial hypertrophy, myocyte disarray, interstitial fibrosis	Identification of pathogenic sarcomeric variants
Dilated cardiomyopathy	*TTN*, *LMNA*, *FLNC*, *DSP*	Ventricular dysfunction and arrhythmias	Ventricular dilatation, fibrosis, myocyte degeneration	Identification of genetic substrate, particularly in young or unexplained cases
Arrhythmogenic cardiomyopathy	*PKP2*, *DSP*, *DSG2*, *DSC2*, *JUP*	Ventricular arrhythmias, SCD	Fibrofatty replacement and/or myocardial fibrosis	Detection of desmosomal and related pathogenic variants
Left ventricular non-compaction cardiomyopathy	*MYH7*, *TAZ*, *ACTC1*	Heart failure and ventricular arrhythmias	Prominent trabeculation and deep intertrabecular recesses	Support of diagnosis in selected cases
Long QT syndrome	*KCNQ1*, *KCNH2*, *SCN5A*	Polymorphic ventricular tachycardia, torsades de pointes	Usually absent	Particularly relevant in autopsy-negative SCD
Brugada syndrome	*SCN5A* and other genes	Ventricular fibrillation and SCD	Usually absent	May identify a molecular substrate in selected cases
Catecholaminergic polymorphic ventricular tachycardia	*RYR2*, *CASQ2*	Exercise-/catecholamine-induced ventricular arrhythmias	Usually absent	Important in young autopsy-negative SCD
Short QT syndrome	*KCNH2*, *KCNQ1*, *KCNJ2*	Atrial and ventricular arrhythmias, SCD	Usually absent	Identification of rare inherited arrhythmic disorders
Other inherited arrhythmic disorders	Multiple genes	Variable	Usually absent or non-specific	May provide a diagnosis when conventional autopsy is negative

**Table 3:** Current approaches to post-mortem genetic testing.

Approach	Main Characteristics	Advantages	Limitations	Potential Application in SCD
Targeted gene panels	Analysis of selected genes associated with inherited cardiac diseases	Cost-effective; relatively rapid; easier interpretation	Limited to predefined genes; may miss novel or unexpected genes	First-line testing in selected SCD cases
Whole-exome sequencing (WES)	Analysis of protein-coding regions	Broader genomic coverage; identification of unexpected variants	Limited assessment of non-coding regions; VUS burden	Unresolved cases and research settings
Whole-genome sequencing (WGS)	Analysis of coding and non-coding genomic regions	Comprehensive genomic assessment; potential detection of structural and regulatory variants	Higher data complexity; interpretation and cost	Increasingly relevant for unresolved cases
Long-read sequencing	Detection of large/complex variants and difficult genomic regions	Improved structural variant and repeat detection	Limited clinical availability; higher cost	Future approach for genetically unresolved SCD
RNA sequencing/transcriptomics	Assessment of gene expression and splicing	Functional evidence for selected variants	RNA degradation post-mortem; tissue-dependent	Functional clarification of variants
Multi-omics	Integration of genomic, transcriptomic, proteomic and metabolomic data	Functional and mechanistic characterization	High complexity; limited standardization	Emerging research strategy
AI-assisted genomic interpretation	Computational prioritization and integration of genomic and phenotypic data	Potentially improves variant prioritization	Requires validated datasets; risk of bias	Emerging adjunct to molecular autopsy

**Table 4:** Framework for genetic variant interpretation in molecular autopsy.

Interpretative Domain	Key Considerations	Relevance to Molecular Autopsy
**Gene–disease validity**	Strength of the association between the gene and the phenotype	Essential prerequisite before attributing SCD to a genetic finding
**Disease mechanism**	Established mechanism, e.g., loss-of-function, gain-of-function, dominant-negative	Determines whether the variant type is biologically plausible
**Variant-level evidence**	Population, functional, segregation, clinical and computational evidence	Determines variant pathogenicity
**Phenotypic concordance**	Compatibility with autopsy and clinical phenotype	Essential for causal inference
**Familial evidence**	Segregation, penetrance and expressivity	Strengthens or weakens causal interpretation

**Table 5:** Clinical and familial implications of molecular autopsy.

Molecular Autopsy Finding	Implications for Relatives	Recommended Approach
Pathogenic/likely pathogenic variant with phenotype concordance	High suspicion of inherited disease	Genetic counselling and cascade screening
Pathogenic/likely pathogenic variant without evident structural phenotype	Possible inherited channelopathy or incompletely penetrant disease	Variant-specific clinical assessment of relatives
VUS	Causality remains uncertain	Do not use VUS alone for predictive clinical decisions
Negative genetic testing	Genetic cause cannot be excluded	Clinical/familial assessment based on phenotype and family history
Multiple potentially relevant variants	Possible oligogenic or modifying effects	Multidisciplinary interpretation
Variant reclassified after initial testing	Previous interpretation may change	Reanalysis and communication when clinically relevant
Familial pathogenic variant identified	At-risk carriers can be identified before symptoms	Cascade testing and cardiological surveillance
No molecular diagnosis but strong family history	Persistent suspicion of inherited disease	Clinical screening of relatives despite negative molecular testing

**Table 6:** Current limitation and future perspectives.

Challenge	Current Limitation	Future Perspective
Diagnostic yield	A substantial proportion of SCD remains genetically unexplained	WGS, long-read sequencing and discovery of new disease genes
Variant interpretation	High number of VUS and incomplete genotype–phenotype correlation	Improved functional studies and gene-/disease-specific frameworks
Pre-analytical factors	Post-mortem degradation and variable sample quality	Standardized sampling and tissue preservation protocols
Protocol heterogeneity	Differences between forensic and clinical centres	International consensus protocols
Multi-omics	Limited availability and post-mortem tissue constraints	Integrated genomic, transcriptomic, proteomic and metabolomic analyses
Artificial intelligence	Limited validation in post-mortem cardiovascular pathology	AI-assisted variant prioritization and multimodal data integration
Data sharing	Rare variants and small individual cohorts	International interoperable databases
Familial management	Variable access to genetic counselling and cascade screening	Structured multidisciplinary pathways
Ethical issues	Privacy, consent and disclosure of post-mortem genetic information	Harmonized ethical and legal frameworks
Long-term data interpretation	Variant classification may change over time	Periodic genomic reanalysis and updated reporting

**Table 7:** From sudden cardiac death to molecular diagnosis and familial prevention.

Step	Investigation	Main Objective	Potential Outcome
1	Clinical and circumstantial assessment	Define the probability of inherited cardiac disease	Identification of cases requiring molecular investigation
2	Complete cardiovascular autopsy	Identify structural causes of SCD	Diagnosis of cardiomyopathy or other structural disease
3	Histopathology	Characterize microscopic myocardial abnormalities	Support for pathological diagnosis
4	Toxicological analysis	Exclude toxicological causes	Differential diagnosis of SCD
5	Molecular autopsy	Identify genetic variants	Molecular diagnosis or genetic risk assessment
6	Variant interpretation	Establish clinical relevance	Pathogenic/likely pathogenic variant, VUS or negative result
7	Genotype–phenotype correlation	Assess causality	Integrated molecular–pathological diagnosis
8	Family evaluation	Identify potentially affected relatives	Identification of individuals at risk
9	Cascade screening	Detect familial carriers	Early diagnosis and preventive management
10	Long-term follow-up and reanalysis	Address evolving genomic knowledge	Updated diagnosis and risk stratification

## Data Availability

No new data were created or analyzed in this study. Data sharing is not applicable to this article.

## Abbreviations

ACM	Arrhythmogenic Cardiomyopathy
ACMG	American College of Medical Genetics and Genomics
AMP	Association for Molecular Pathology
ARVC	Arrhythmogenic Right Ventricular Cardiomyopathy
BrS	Brugada Syndrome
CPVT	Catecholaminergic Polymorphic Ventricular Tachycardia
DCM	Dilated Cardiomyopathy
ECG	Electrocardiogram
FFPE	Formalin-Fixed, Paraffin-Embedded
HCM	Hypertrophic Cardiomyopathy
LQTS	Long QT Syndrome
MA	Molecular Autopsy
NGS	Next-Generation Sequencing
P/LP	Pathogenic/Likely Pathogenic
SCD	Sudden Cardiac Death
SCDY	Sudden Cardiac Death in the Young
SQTS	Short QT Syndrome
SUD	Sudden Unexplained Death
VUS	Variant of Uncertain Significance
WES	Whole-Exome Sequencing
WGS	Whole-Genome Sequencing
